# Synthesis and characterization of coumarin-derived sulfur analogues using Lawesson’s reagent

**DOI:** 10.1107/S2053229625000956

**Published:** 2025-02-26

**Authors:** Neliswa Mama, Stiaan Schoeman, Lisa Myburgh, Eric C. Hosten

**Affiliations:** a Department of Chemistry, Nelson Mandela University, PO Box 77000, Port Elizabeth 6031, South Africa; University of the Witwatersrand, South Africa

**Keywords:** coumarin, thio­nation, UV–Vis absorption, crystal structure, Hirshfeld surface, energy framework, topology, Lawesson’s reagent, Knoevenagel, chemosensor

## Abstract

The synthesis and characterization of six novel coumarin derivatives containing O and S atoms are described here, namely, ethyl 2-oxo-2*H*-chro­mene-3-car­box­yl­ate, C_12_H_10_O_4_ (**S1a**), ethyl 2-sul­fan­yl­idene-2*H*-chro­mene-3-car­box­yl­ate, C_12_H_10_O_3_S (**S2a**), ethyl 2-sul­fan­yl­idene-2*H*-chro­mene-3-carbo­thio­ate, C_12_H_10_O_2_S_2_ (**S3a**), ethyl 8-meth­oxy-2-oxo-2*H*-chro­mene-3-car­box­yl­ate, C_13_H_12_O_5_ (**S1b**), ethyl 8-meth­oxy-2-sul­fan­yl­idene-2*H*-chro­mene-3-car­box­yl­ate, C_13_H_12_O_4_S (**S2b**), and ethyl 8-meth­oxy-2-sul­fan­yl­idene-2*H*-chro­mene-3-carbo­thio­ate, C_13_H_12_O_3_S_2_ (**S3b**). Compounds **S1a/b** were synthesized in good yields following the Knoevenagel con­densation method. The thio­carbonyl analogues of these com­pounds, **S2a/b** and **S3a/b**, were obtained using Lawesson’s reagent as a thio­nating com­pound. The structures of **S2a/b** and **S3a/b** were confirmed using FT–IR, ^1^H and ^13^C NMR, and UV–Vis spectroscopy, and single-crystal X-ray diffraction. Hirshfeld surface and energy framework analyses show that stacked π–π ring inter­actions occur for all the structures obtained here, and various hy­dro­gen-bond inter­actions link the stacks to form three-dimensional energy frameworks.

## Abstract 2

The synthesis and characterization of six novel coumarin derivatives containing O and S atoms are described. Hirshfeld surface and energy framework analyses show that stacked π–π ring inter­actions occur for all the structures and various hy­dro­gen-bond inter­actions link the stacks to form three-dimensional energy frameworks.

## Introduction

**Figure 1:**
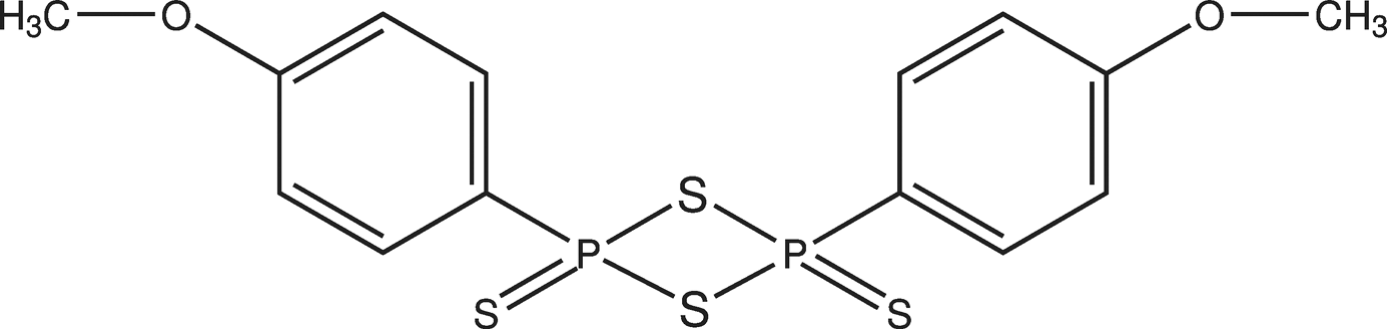
The mol­ecular structure of Lawesson’s reagent.

Coumarin derivatives are heterocyclic com­pounds that occur naturally and were first isolated from tonka beans in 1820 by Vogel (Matos *et al.*, 2015 ▸). Various reactions can be used to synthesize coumarin-based com­pounds. These reactions include the Perkin, Claisen, Pechmann, Heck lactonization, Baylis–Hillman, Michael, Wittig, Knoevenagel, Reformatsky and Kostanecki reactions. Coumarin derivatives are essential due to their various uses, such as photosensitizers, fluorescent materials, optical brighteners, laser dyes and pharmaceuticals (Liu *et al.*, 2012 ▸; Bakhtiari *et al.*, 2014 ▸; Zhang *et al.*, 2016 ▸; Abdallah *et al.*, 2020 ▸). In addition, these com­pounds possess desirable characteristics which include good spectral properties, the ability to undergo multiple substitution reactions offering versatility in chemical modification and the ability to carry out electrophilic substitution reactions to obtain various coumarin derivatives (Bojtár *et al.*, 2019 ▸; Olson *et al.*, 2013 ▸; Hansen *et al.*, 2015 ▸). Lawesson’s reagent (Fig. 1 ▸) is a sulfur-rich thio­nating reagent that can be used in the conversion of carbonyl oxygen to form the corresponding thio­carbonyl analogues (Kayukova *et al.*, 2015 ▸; Jesberger *et al.*, 2003 ▸; Khatoon & Abdulmalek, 2021 ▸).

The com­pounds ethyl 2-oxo-2*H*-chro­mene-3-car­box­yl­ate (**S1a**), ethyl 2-sul­fan­yl­idene-2*H*-chro­mene-3-car­box­yl­ate (**S2a**), ethyl 2-sul­fan­yl­idene-2*H*-chro­mene-3-carbo­thio­ate (**S3a**), ethyl 8-meth­oxy-2-oxo-2*H*-chro­mene-3-car­box­yl­ate (**S1b**), ethyl 8-meth­oxy-2-sul­fan­yl­idene-2*H*-chro­mene-3-car­box­yl­ate (**S2b**) and ethyl 8-meth­oxy-2-sul­fan­yl­idene-2*H*-chro­mene-3-carbo­thio­ate (**S3b**) (Scheme 1) were prepared and characterized.

## Experimental

### Materials and procedures

The chemicals that were used in the synthesis and analysis of com­pounds **S1**–**S3** were purchased from Sigma Merck and were used without purification. The synthesis reactions were monitored using thin-layer chromatography (TLC) and nuclear magnetic resonance (NMR) and IR spectroscopy. The TLC plates that were used to monitor the reactions were aluminium sheets coated with silica gel 60 F254 and were viewed under UV light to confirm the formation of various products. The relevant NMR samples were prepared using CDCl_3_ with tetra­methyl­silane (TMS) as an inter­nal reference. The NMR chemical shifts are recorded in parts per million (ppm) and the coupling constants are recorded in Hz.
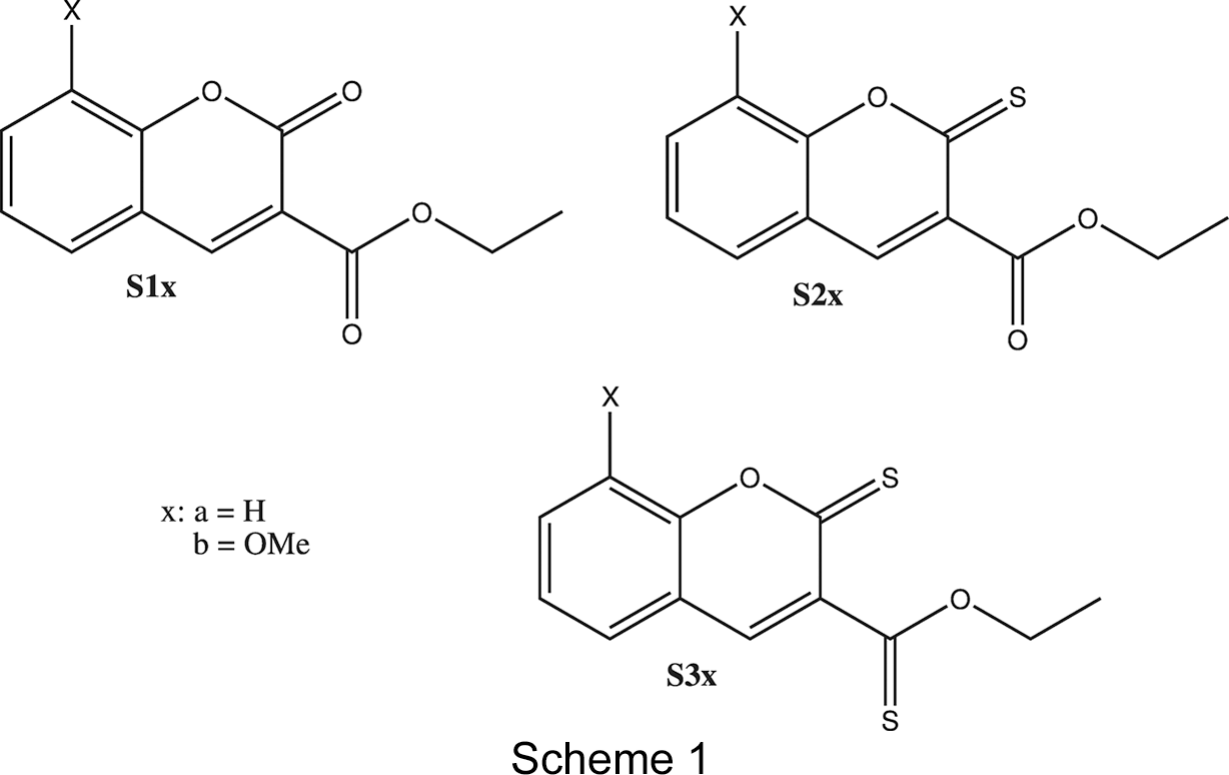


### Synthesis of coumarin derivatives S1 (Patil *et al.*, 2011 ▸)

Compounds **S1a** and **S1b** were prepared by refluxing a mixture containing equimolar qu­anti­ties of diethyl malonate (0.01 *M*) and a salicyl­aldehyde derivative (0.01 *M*) in ethanol (25 ml) in the presence of 1 ml of piperidine and 5 drops of glacial acetic acid for 3 h. The mixture was placed on ice and the resulting precipitate was filtered off, washed with ice-cold ethanol and dried to yield **S1a** as a white solid and **S1b** as a yellow solid.

Analytical data for **S1a**: yield 95%. ^1^H NMR (CDCl_3_): δ 1.33–1.37 (*t*, 3H), 3.91 (*s*, 3H), 4.32–4.37 (*q*, 2H), 7.12–7.22 (*m*, 3H), 8.43 (*s*, 1H). ^13^C NMR (CDCl_3_): δ 14.18, 56.27, 61.84, 115.86, 118.29, 118.35, 120.60, 124.74, 144.70, 146.94, 148.74, 156.12, 162.92. IR ν_max_ (cm^−1^): 3040–2854 (C—H), 1735 (C=O), 1701 (C=O).

Analytical data for **S1b**: yield 47%. ^1^H NMR: (CDCl_3_): δ 1.42–1.45 (*t*, 3H), 4.41–4.47 (*q*, 2H), 7.34–7.39 (*m*, 2H), 7.63–7.69 (*m*, 2H), 8.54 (*s*, 1H). ^13^C NMR (CDCl_3_): δ 14.24, 62.00, 116.82, 117.92, 118.42, 124.84, 129.49, 134.32, 148.57, 155.21, 156.71, 163.10. IR ν_max_ (cm^−1^): 3065–2914 (C—H), 1761 (C=O). M.p. 93–95 °C.

### Preparation of S2 and S3

A mixture of coumarin derivative **S1a** or **S1b** and Lawesson’s reagent (LR) was added in a 1:2 molar ratio to dry toluene (25 ml). The reaction mixture was refluxed under an N_2_ atmosphere for 8 h (Scheme 2). The resulting solution was added to water and extracted three times using ethyl acetate. The extracts were washed with brine followed by water and dried using anhydrous Na_2_SO_4_. The ethyl acetate was re­moved under reduced pressure and the products were purified using preparative TLC (DCM–PET ether solvent).
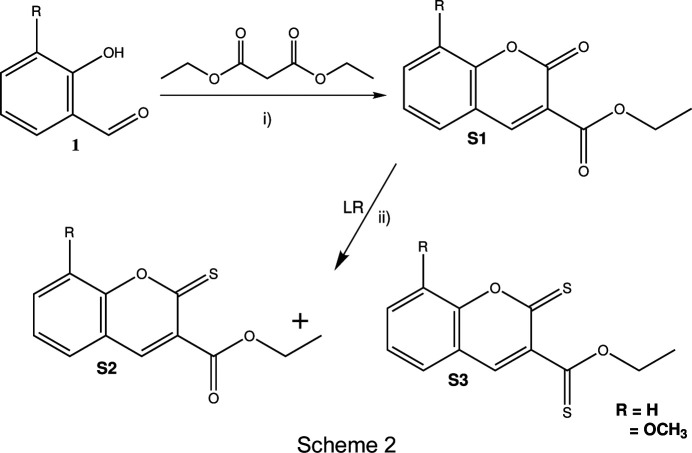


Analytical data for **S2a**, ^1^H NMR (CDCl_3_): δ 1.41–1.45 (*t*, 3H), 4.01 (*s*, 3H), 4.41–4.44 (*q*, 2H), 7.14–7.19 (*m*, 2H), 7.29–7.31 (*m*, 1H), 7.85 (*s*, 1H). ^13^C NMR (CDCl_3_): δ 14.06, 56.32, 62.24, 115.13, 119.78, 120.13, 125.69, 132.54, 135.72, 146.61, 146.98, 164.94, 191.38. IR ν_max_ (cm^−1^): 3022–2852 (C—H), 1728 (C=O).

Analytical data for **S2b**, ^1^H NMR (CDCl_3_): δ 1.42–1.45 (*t*, 3H), 4.41–4.47 (*q*, 2H), 7.36–7.39 (*t*, 1H), 7.48–7.61 (d-d, 2H), 7.66–7.70 (*t*, 1H), 7.88 (*s*, 1H). ^13^C NMR (CDCl_3_): δ 14.06, 62.28, 116.58, 119.44, 125.73, 128.76, 132.31, 133.78, 135.62, 157.01, 164.89, 192.16. IR ν_max_ (cm^−1^): 3055–2930 (C—H), 1718 (C=O). M.p. 80–84 °C.

Analytical data for **S3a**, ^1^H NMR (CDCl_3_): δ 1.51–1.54 (*t*, 3H), 4.01 (*s*, 3H), 4.71–4.76 (*q*, 2H), 7.13–7.16 (*m*, 2H), 7.28–7.29 (*m*, 1H), 7.74 (*s*, 1H). ^13^C NMR (CDCl_3_): δ 13.33, 25.32, 69.54, 114.53, 119.67, 120.69, 125.59, 133.97, 141.33, 146.61, 146.74, 191.63, 211.05. IR ν_max_ (cm^−1^): 3015–2845 (C—H).

Analytical data for **S3b**, ^1^H NMR (CDCl_3_): δ 1.51–1.55 (*t*, 3H), 4.71–4.77 (*q*, 2H), 7.34–7.38 (*t*, 1H), 7.48–7.60 (*dd*, 2H), 7.62–7.66 (*t*, 1H), 7.77 (*s*, 1H). ^13^C NMR (CDCl_3_) δ_C_: 13.33, 69.56, 116.48, 119.97, 119.97, 125.64, 128.56, 133.06, 133.87, 141.11, 156.11, 192.40, 210.96. IR ν_max_ (cm^−1^): 3050–2840 (C—H). M.p. 77–82 °C.

### Crystallization, refinement and analyses of the structures

**Figure 2:**
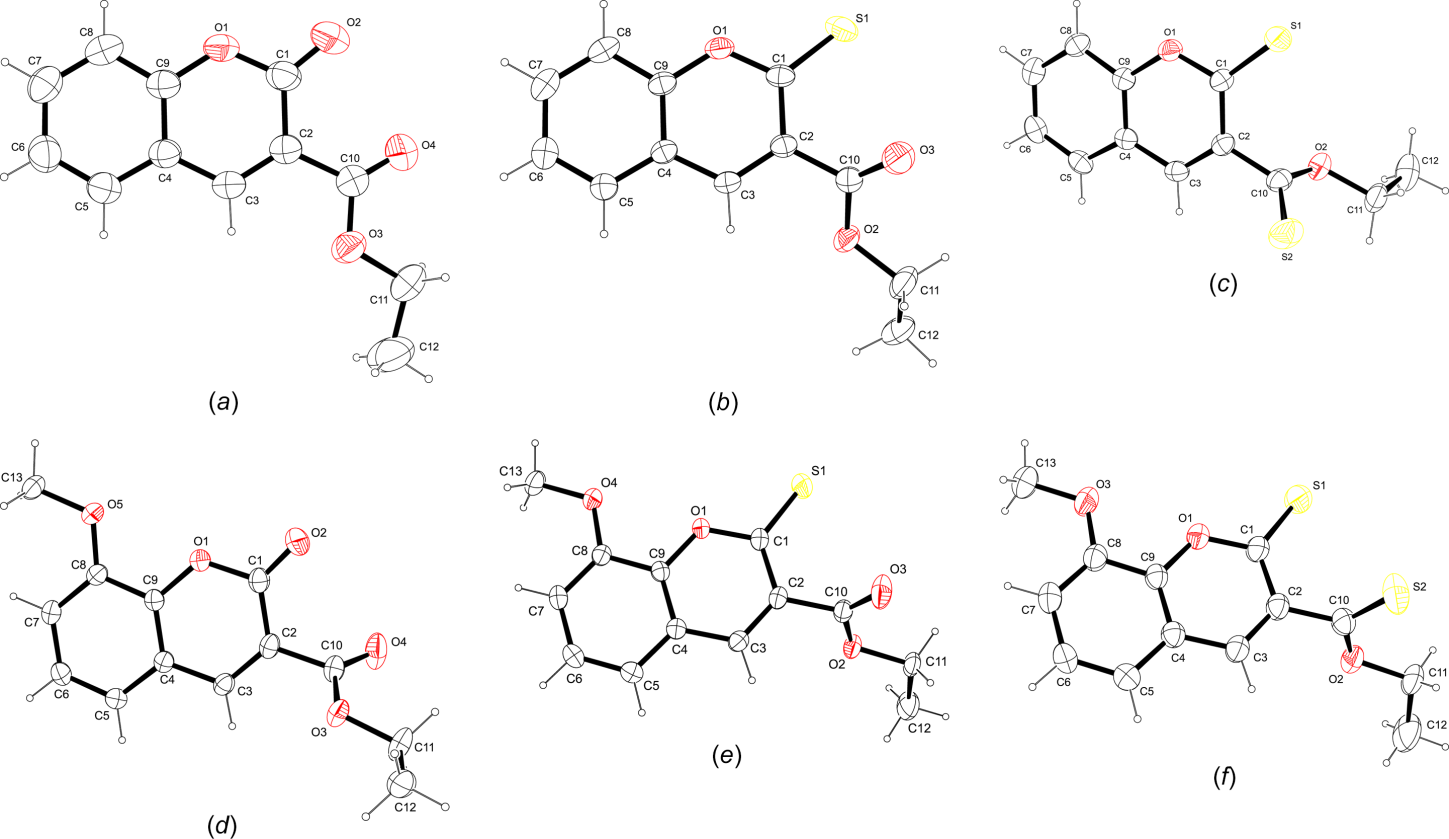
The mol­ecular structures of (*a*) **S1a**, (*b*) **S2a**, (*c*) **S3a**, (*d*) **S1b**, (*e*) **S2b** and (*f*) **S3b**, showing the atom-labelling schemes. Displacement ellipsoids are drawn at the 50% probability level.

Growing crystals for diffraction studies was achieved by slow vaporation from hexane. Crystal data, data collection and structure refinement details are summarized in Table 1 ▸. All H atoms were placed in calculated positions and refined using a riding-model approximation, with *U*_iso_(H) = 1.2*U*_eq_(C). The H atoms of the methyl groups were allowed to rotate with a fixed angle around the C—C bonds to best fit the experimental electron density, with *U*_iso_(H) = 1.5*U*_eq_(C). Structure **S3b** was refined as an inversion twin. Fig. 2 ▸ shows the mol­ecular structures obtained (Farrugia, 2012 ▸). *CrystalExplorer* (Spackman *et al.*, 2021 ▸) was utilized to investigate the inter­molecular inter­actions using the Hirshfeld surfaces, fingerprint plots, energy frameworks and lattice energies (Tan *et al.*, 2019 ▸; Mackenzie *et al.*, 2017 ▸). The basis set B3LYP/631-G(d,p) was used for all calculations. The topologies of the electrostatic and van der Waals inter­actions were determined with *TopCryst* (Shevchenko & Blatov, 2021 ▸).

## Results and discussion

### Synthesis of coumarin derivatives S1–S3

The synthesis of coumarin ester derivatives **S1a/b** was achieved using the Knoevenagel con­densation method, as outlined in Scheme 2. The reaction between coumarin ester derivatives and an excess of Lawesson’s reagent was con­ducted in toluene under nitro­gen gas to obtain thio­carbonyl analogues **S2** (major product) and **S3** (minor product) in good yields. The structures of these analogues were confirmed by FT–IR, ^1^H NMR and ^13^C NMR spectroscopic and X-ray crystallographic data.

### UV–Vis absorption analysis of coumarin derivatives S1–S3

**Figure 3:**
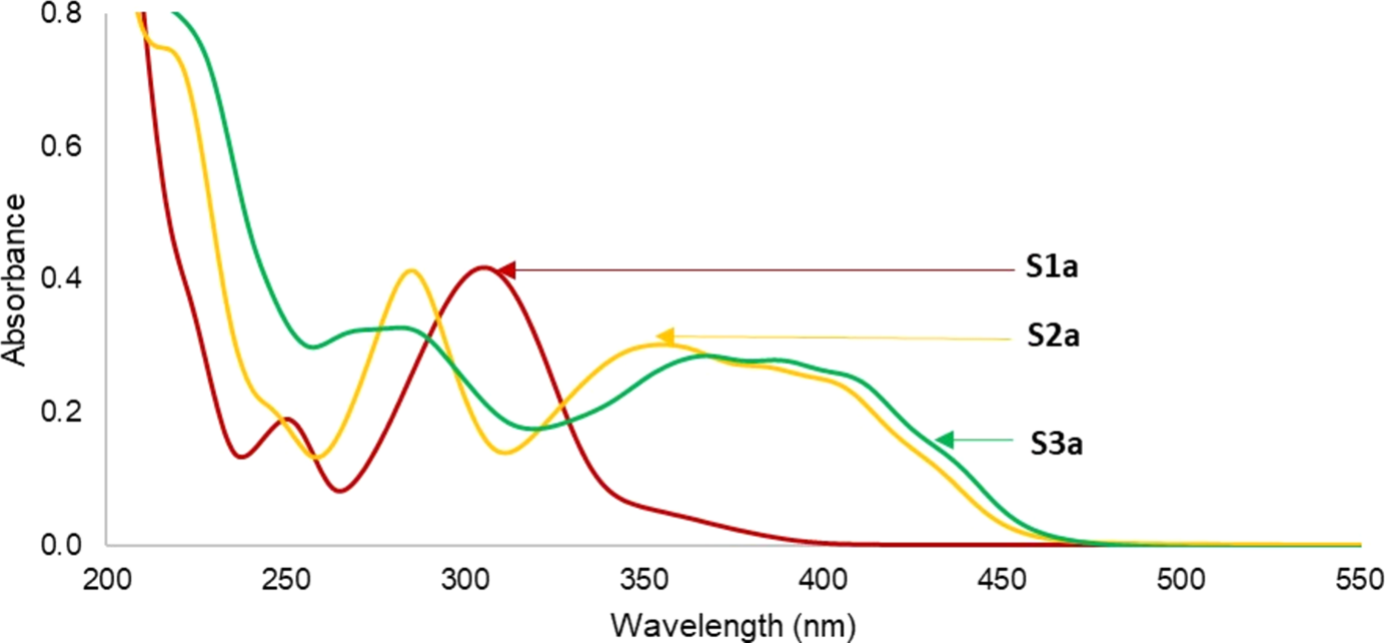
UV–Vis spectra of com­pounds **S1a**, **S2a** and **S3a** in aceto­nitrile medium.

The absorption spectra of the coumarin derivatives **S1a/b**, **S2a/b** and **S3a/b** were obtained in aceto­nitrile and displayed absorbance properties in the region of 238–460 nm (Figs. 3 ▸ and 4 ▸). The absorption spectra of **S1a** and **S1b** are characterized by intense absorption peaks near 270–350 nm, which could be attributed to π–π* transitions from the conjugated coumarin ring, whereas the less intense bands around 238–260 nm for **S1a** and at 272–341 nm for **S1b** were attributed to n–π from the carbonyl groups on the coumarin ring. The presence of the strong electron-donating group at position 8 in com­pound **S1b** seems to increase the electron density of the coumarin ring; thus, the π–π* transition of the mol­ecule reflected in the observed less intense band at lower wavelength (242–255 nm).

On the other hand, the thio­carbonyl analogues **S2a/b** and **S3a/b** showed two strong absorption bands around 260–340 and 345–450 nm. The presence of the S atoms in com­pounds **S2** and **S3** resulted to a new band at 345–450 nm which is attributed to n–π* transitions from the S atoms.

### Crystal structures

The title com­pounds crystallized in monoclinic space groups, except for **S3b**, which crystallized in an ortho­rhom­bic space group. All the bond lengths and angles are in the expected ranges. A search of the Cambridge Structural Database (CSD, Version 5.45, update of June 2024; Groom *et al.*, 2016 ▸) yielded only four crystal structures of **S1a** and **S1b** (García-Báez *et al.*, 2003 ▸; Mahendra *et al.*, 2003 ▸; Shang *et al.*, 2015 ▸; Takahashi *et al.*, 2006 ▸) identical to the structures reported here.

**Figure 5:**
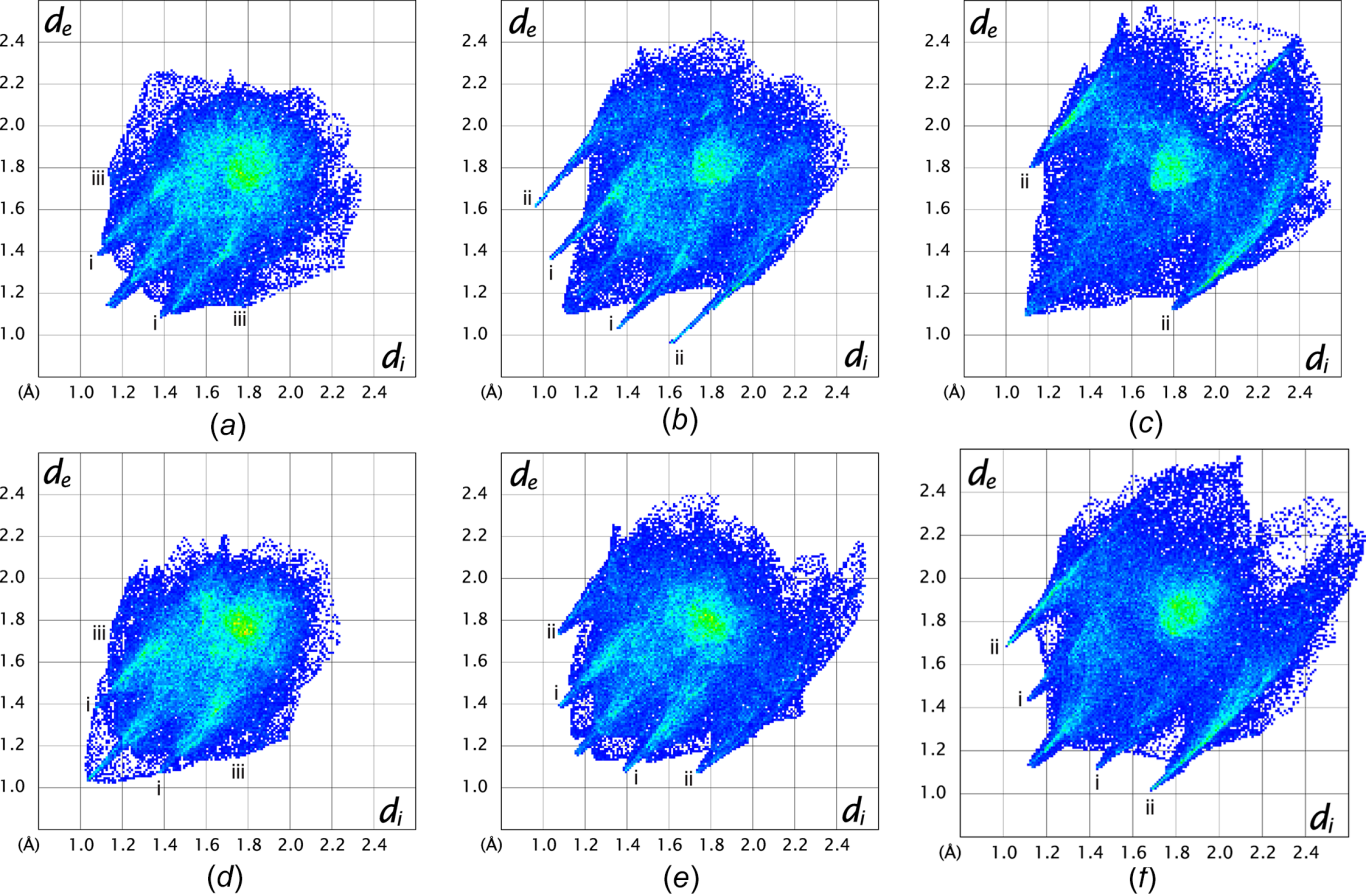
Hirshfeld fingerprint plots of (*a*) **S1a**, (*b*) **S2a**, (*c*) **S3a**, (*d*) **S1b**, (*e*) **S2b** and (*f*) **S3b**. Spike indicators: (i) O⋯H/H⋯O; (ii) S⋯H/H⋯S; (iii) C⋯H/H⋯C.

**Figure 6:**
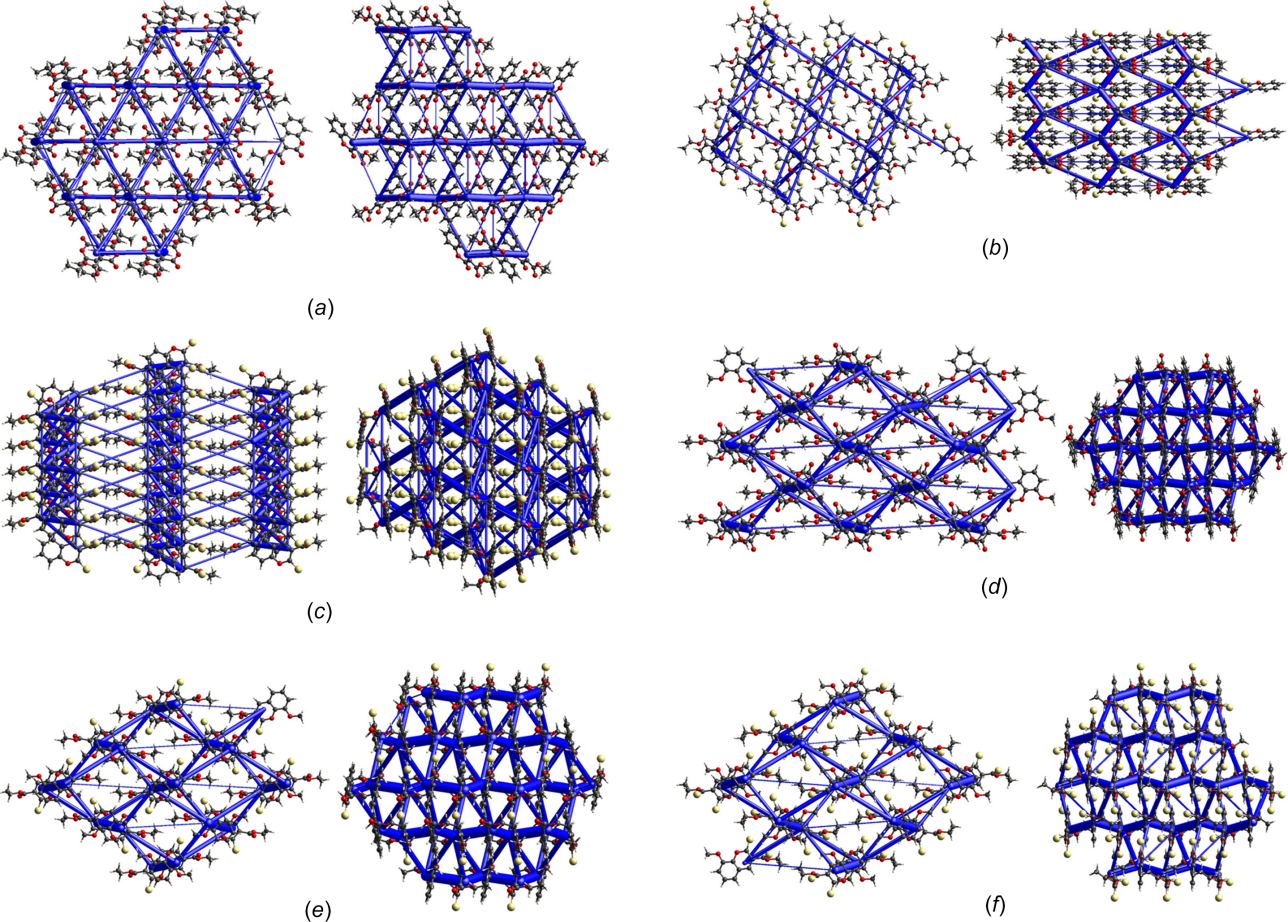
Energy frameworks within a 10 Å sphere and with a tube size of 100 and a cut-off at 5 kJ mol^−1^. (*a*) **S1a** viewed down the *a* and *c* axes; (*b*) **S2a** viewed down the *b* and *c* axes; (*c*) **S3a** viewed down the *a* and *c* axes; (*d*) **S1b** viewed down the *a* and *c* axes; (*e*) **S2b** viewed down the *a* and *c* axes; (*f*) **S3b** viewed down the *a* and *c* axes.

**Figure 7:**
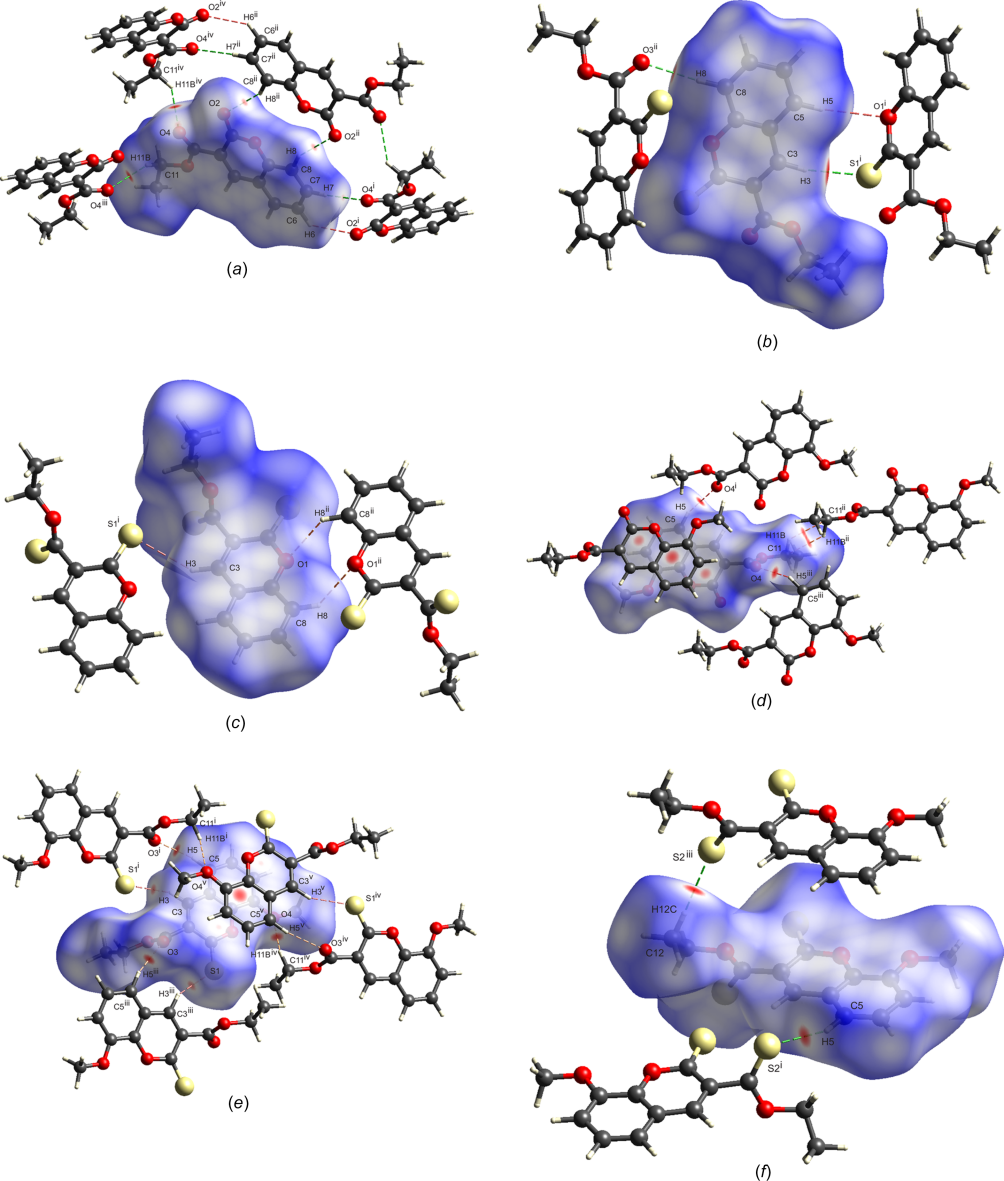
Hirshfeld *d*_norm_ surface and selected inter­molecular hy­dro­gen-bond inter­actions. For clarity, not all inter­actions are shown. [Symmetry codes for **S1a**: (i) −*x*, *y* + 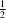, −*z* + 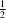; (ii) −*x*, −*y* + 1, −*z*; (iii) *x*, −*y* + 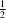, *z* + 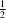; (iv) *x*, −*y* + 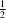, *z* − 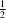; for **S2a**: (i) *x*, −*y* + 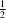, *z* − 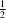; (ii) −*x* + 1, *y* + 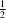, −*z* + 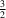; for **S3a**: (i) *x*, *y* − 1, *z*; (ii) −*x* + 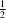, −*y* + 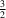, −*z* + 1; for **S1b**: (i) −*x* + 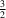, *y* + 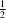, −*z* + 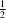; (ii) −*x* + 1, −*y* + 1, −*z*; (iii) −*x* + 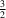, *y* − 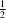, −*z* + 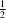; for **S2b**: (i) −*x* + 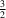, *y* − 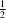; (iii) −*x* + 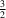, *y* + 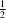, −*z* + 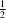; (iv) *x* + 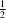, −*y* + 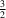, *z* + 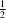; (v) −*x* + 2, −*y* + 1, −*z* + 1; for **S3b**: (i) −*x* + 1, *y* + 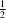, −*z* + 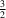; (iii) *x* − 1, *y*, *z*.

The structure of **S1a** is essentially planar, with the O2 and C10 atoms 0.279 (2) and 0.170 (2) Å in opposite directions out of the mean coumarin plane and the mean plane through C10/O3/O4 rotated by 10.7 (2)° from the coumarin plane. There is one intra­molecular inter­action, *i.e.* C3—H3⋯O3 (Table 2 ▸). The energy framework calculations show that the strongest inter­actions are due to centrosymmetric π-ring offset-stacked chains down the *a* axis and with total energy contributions of −55.91 and −48.0 kJ mol^−1^ (dispersion contributions are −61.3 and −64.8 kJ mol^−1^, respectively). The distance between the mean coumarin planes alternates between 3.18 and 3.46 Å. The −55.9 kJ mol^−1^ inter­actions also include the C11—H11*A*⋯π-ring inter­action with the C3–C9 ring, which is visible on the fingerprint plot as a broad wing, but obscured by the H⋯H contact surface [Fig. 5 ▸(*a*)]. Each π-stack is linked to six other stacks with inter­actions of total energies of −31.0 or −21.8 kJ mol^−1^. The electrostatic inter­actions occur in all dimensions, while the dispersion inter­actions are in planes parallel to the *ac* plane. As a result, the total energy framework [Fig. 6 ▸(*a*)] extends in all dimensions, with the underlying net determined as **16T3** by *TopCryst*. Centrosymmetric pairs of C8—H8⋯O2^ii^ inter­actions link two mol­ecules with an 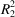
(12) graph-set motif (Bernstein *et al.*, 1995 ▸). This corresponds to the −31.0 kJ mol^−1^ inter­action linking π-stacks. The C6—H6⋯O2^i^ and C7—H7⋯O4^i^ inter­actions [Fig. 7 ▸(*a*) and Table 2 ▸] link mol­ecules with *C*(8) and *C*(9) descriptors, respectively, parallel to the *b* axis, with the mol­ecules arranged in a zigzag chain fashion. This corresponds to the −21.8 kJ mol^−1^ inter­action. The dominant inter­molecular hy­dro­gen-bond inter­action, as shown by the *d*_norm_ Hirshfeld surface, is C11—H11*B*⋯O4^iii^, which links mol­ecules alternately in two planes parallel to the *c* axis, with a dihedral angle of 60.6° between the planes. This inter­action can be described with a *C*(5) descriptor and is visible on the fingerprint plot as a broad spike [Fig. 6 ▸(*a*)]. The energy framework inter­action is only −7.5 kJ mol^−1^ in this orientation and is mainly dispersion in nature.

For **S2a**, the S1 and C10 atoms are 0.203 (1) and 0.210 (2) Å, respectively, in opposite directions out of the mean coumarin ring, and with the mean plane of the C10/O3/O2 car­box­yl­ate group rotated by 38.7 (1)° from the plane of the coumarin ring. The mol­ecules are arranged in zigzag planes parallel to the *ac* plane and centrosymmetric offset π-ring chains stack parallel to the *b* axis, with alternating distances of 3.41 and 3.09 Å between the mean coumarin planes. The energy frameworks show the strongest inter­actions down the *b* axis, with alternating total energies of −48.9 and −35.1 kJ mol^−1^. The total energy framework [Fig. 6 ▸(*b*)] extends in all dimensions, with the main inter­actions in the *bc* plane. The underlying net was determined as **bcu-x** or **sqc38by** by *TopCryst*. Adjacent stacks are linked with inter­actions of −31.0 kJ mol^−1^ total energy, with −20.4 and −21.2 kJ mol^−1^ electrostatic and dispersion contributions, respectively. This arises from the C8—H8⋯O3^ii^ chain inter­actions [Fig. 7 ▸(*b*)], with a *C*(8) descriptor, seen as a sharp peak on the fingerprint plot [Fig. 5 ▸(*b*)]. Also, the Hirshfeld shape index indicates a prominent C1=S1⋯π ring inter­action with the C4–C9 ring, with a herringbone packing orientation. The shortest S⋯centroid distance is 3.7011 (8) Å. Prominent on the Hirshfeld *d*_norm_ surface is the C3—H3⋯S1^i^ chain inter­action down the *c* axis, with a *C*(5) descriptor and a sharp peak on the fingerprint plot [Fig. 5 ▸(*b*)]. This corresponds to the −20.0 kJ mol^−1^ energy framework inter­action, with electrostatic and dispersion contributions of −16.7 and −17.6 kJ mol^−1^, respectively. The shape index surface also shows weaker C5—H5⋯O1^i^ and C12—H12*C*⋯S1^i^ contributions (Table 3 ▸). The C12—H12*A*⋯S1^iv^ hydrogen bond is also prominent on the shape index linking adjacent chains that correspond to the −19.9 kJ mol^−1^ inter­action on the energy framework. Also contributing is the weak C12—H12*B*⋯O3^iii^ inter­action with a *C*(6) descriptor.

In **S3a**, com­pared to **S1a** and **S2a**, the eth­oxy­carbonyl group is rotated most from the mean coumarin plane, by 75.41 (5)°. Atoms S1 and C10 are only 0.026 (2) and 0.030 (2) Å out of this plane. The *d*_norm_ surface for **S3a** is featureless [Fig. 7 ▸(*c*)]; however, the shape index indicates a number of possible inter­actions. On either side of the coumarin ring there are stacked centrosymmetrical π-ring inter­actions with alternating coumarin planes separated by 3.35 and 3.40 Å. The stacking occurs in the [110] and [1
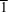
0] directions in planes parallel to the *ab* plane. The total energy alternates between −45.7 and −47.8 kJ mol^−1^ down the stack. Stacks in the *ab* plane are linked, with inter­actions having total energies of −14.7 and −23.0 kJ mol^−1^, and the stack planes are joined with inter­actions of −13.4 kJ mol^−1^. The total energy framework [Fig. 6 ▸(*c*)] extends in all dimensions and the underlying topology determined by *TopCryst* is **tcf-x**. The shortest inter­action is C3—H3⋯S1^i^ [Fig. 7 ▸(*c*)], with a length of 3.03 Å. This links mol­ecules down the *b* axis, with a *C*(5) descriptor, results in the only noticeable spike on the fingerprint plot [Fig. 5 ▸(*c*)] and corresponds to the −14.7 kJ mol^−1^ inter­action. On each edge of the coumarin group there are two inter­actions, both of total energy −23.0 kJ mol^−1^, which include centrosymmetric pairs of C8—H8⋯O1^ii^, and C—H⋯π ring inter­actions in­cluding C8—H8, C3—H3 and C5—H5. The ethyl group is held in place by a number of C—H⋯π⋯S inter­actions, namely, <!?up><!?tlsb><!?down>C12—H12*A*⋯S1^iii^, C12—H12*C*⋯S2^iv^ and C12—H12*B*⋯S2^v^, with lengths varying from 3.15 to 3.33 Å (Table 4 ▸). The C12—H12*A*⋯S1^iii^ inter­action contributes to the −13.4 kJ mol^−1^ inter­action linking planes of stacks together and is mainly dispersion in nature. The fingerprint plot for **S3a** has broad wings for the O⋯H/H⋯O inter­actions which are obscured by the H⋯H contact surface.

The eth­oxy­carbonyl group of **S1b** is rotated to a larger extent [58.27 (4)°] from the coumarin plane com­pared to **S1a**, while the meth­oxy group is only rotated by 7.17 (8)°. In **S1b**, the O2, C10 and O5 atoms are all less that 0.06 Å out of the mean coumarin plane. The centrosymmetric staggered π-ring stacking is prominent down the *a* axis, with alternating layers of 3.28 and 3.43 Å between the mean coumarin planes; this is also evident on the *d*_norm_ surface. The dispersion energy (−74.2 kJ mol^−1^) is dominant, with total energies of −63.9 and −54.9 kJ mol^−1^. Adjacent π-ring stacks are linked on four sides, with inter­actions having total energies of −31.2 and −33.6 kJ mol^−1^, and two other π-ring stacks with inter­actions of −11.6 kJ mol^−1^. A three-dimensional framework [Fig. 6 ▸(*d*)] occurs with a **16-c** net topology. The *d*_norm_ surface shows one strong intra­molecular inter­action C5—H5⋯O4^i^ [Fig. 7 ▸(*d*) and Table 5 ▸] linking atoms in a chain down the *b* axis, with a *C*(7) descriptor and corresponding to the −31.2 kJ mol^−1^ inter­action. This inter­action is also indicated as a spike on the fingerprint plot [Fig. 5 ▸(*d*)], but is obscured by the H⋯H contact surface. The shape index surface also shows evidence of C11—H11*B*⋯H11*B*—C11 π-inter­actions between the methyl­ene C atom of the ethyl group corresponding to the −11.6 kJ mol^−1^ inter­action, which has mainly a dispersion contribution. The −33.6 kJ mol^−1^ inter­action, with both electrostatic and dispersion contributions, arises from a number of C—H⋯O π-inter­actions involving the meth­oxy and eth­oxy groups with atoms O1, O2, O3 and O5. The fingerprint plot has broad wings for the C⋯H/H⋯C contact (obscured by the H⋯H surface) that correspond to C13—H13*B*⋯C10 π-inter­actions between mol­ecules in the π-stack.

In the **S2b** structure, the C10 and S1 atoms lie more than 0.1 Å from the mean coumarin plane, and the plane of the eth­oxy­carbonyl group is rotated by 65.67 (5)°. The meth­oxy group is rotated by 6.53 (10)° with respect to the coumarin plane. Offset π-ring inter­actions are also prominent, with pairs of centrosymmetric inter­actions stacking mol­ecules down the *a* axis in alternating planes 3.31 and 3.37 Å apart. The energy framework calculations show strong dispersion effects of −84.2 and −75.6 kJ mol^−1^ alternating down the π-stacked rings (total energy −60.9 and −67.1 kJ mol^−1^, respectively). The π-ring stacks are connected to four adjacent stacks, with inter­actions having total energies of −30.4 or −32.7 kJ mol^−1^, resulting in a three-dimensional energy framework [Fig. 6 ▸(*e*)], with the underlying topology determined by *TopCryst* as a **16-c** net. Adjacent meth­oxy and eth­oxy­carbonyl groups are linked by a C11—H11*B*⋯O4^ii^ inter­action [Fig. 7 ▸(*e*)], linking mol­ecules in the [101] direction with a *C*(10) motif and corresponding to the −32.7 kJ mol^−1^ inter­action, which has −21.5 and −24.36 kJ mol^−1^ electrostatic and dispersion con­tri­butions, respectively. The shape index indicates possible π-inter­action of the eth­oxy group with atom S1, and of the meth­oxy group with atoms S1 and O2. The *d*_norm_ surface [Fig. 7 ▸(*e*)] shows a prominent inter­molecular C5—H5⋯O3^i^ inter­action that, together with the C3—H3⋯S1^i^ inter­action, links two mol­ecules in a ring 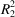
(10) motif (Table 6 ▸). Both these inter­actions cause noticeable spikes on the fingerprint plot [Fig. 5 ▸(*e*)]. Furthermore, these two inter­actions form a chain of inter­actions with *C*(7) and *C*(5) descriptors, respectively, that link mol­ecules down the *b* axis. These inter­actions correspond to the −30.4 kJ mol^−1^ inter­action, with −21.9 and −25.0 kJ mol^−1^ electrostatic and dispersion contributions, respectively.

For **S3b**, the S1 and C10 atoms both lie more than 0.09 Å in opposite directions out of the mean coumarin plane. The eth­oxy­carbonyl and meth­oxy groups are rotated by 73.56 (11) and 1.84 (5)°, respectively, from the mean coumarin plane. There is one intra­molecular C11—H11*B*⋯S2 inter­action of length 2.67 Å. Unlike the previous structures, where the stacked coumarin groups are centrosymmetric and their mean planes parallel, the coumarin groups in **S3c** have twofold screw-axis symmetry and an angle of 4.30° between the successive mean coumarin planes. The coumarin rings are stacked down the *a* axis, with centroid-to-centroid distances of 3.60 Å. The total energy of the inter­action is −58.9 kJ mol^−1^, with dispersion and electrostatic contributions of −69.0 and −20.4 kJ mol^−1^, respectively. There is a prominent C12—H12*C*⋯S2^iii^ inter­action [Fig. 7 ▸(*f*)], with a *C*(6) motif, that links alternate mol­ecules in the π-ring stack along the *a* axis, contributing to the electrostatic contribution and indicated by sharp spikes on the fingerprint plot [Fig. 5 ▸(*f*)]. Electrostatic and dispersion contributions with total energies of −12.2, −24.2 and −32.5 kJ mol^−1^ link four adjacent π-stacks, creating a three-dimensional framework [Fig. 6 ▸(*f*)] with an **18-c** net topology. The C5—H5⋯S2^i^ inter­action links mol­ecules down the *b* axis with a *C*(7) graph-set descriptor. This corresponds to the −24.2 kJ mol^−1^ inter­action, with −19.2 and −25.38 kJ mol^−1^ electrostatic and dispersion contributions, respectively. The −32.5 kJ mol^−1^ framework inter­action has electrostatic and dispersion contributions of −22.5 and −20.3 kJ mol^−1^, respectively. The shape index indicates that this arises from C11—H11*A*⋯O3^ii^, C12—H12*B*⋯S1^ii^ and C13—H13*C*⋯S1^iv^ inter­actions that link mol­ecules down the *c* axis (Table 7 ▸). The C11—H11*A*⋯O3 inter­action has a noticeable spike on the fingerprint plot [Fig. 5 ▸(*f*)]. The remaining inter­action of −12.1 kJ mol^−1^ arises from inter­actions between atom S2 and the meth­oxy group.

**Figure 8:**
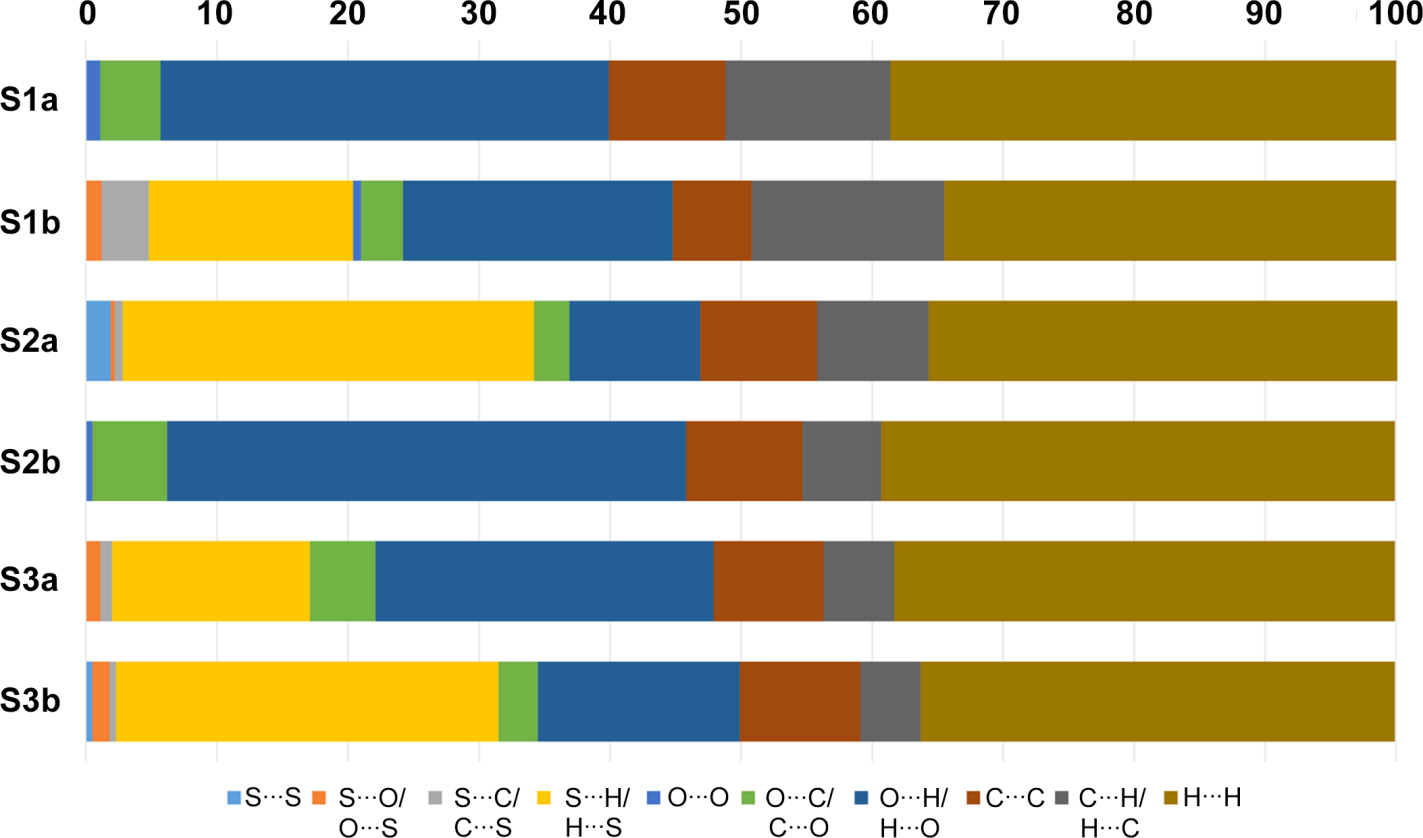
Relative percentage contributions of close contacts to the Hirshfeld surfaces.

**Figure 9:**
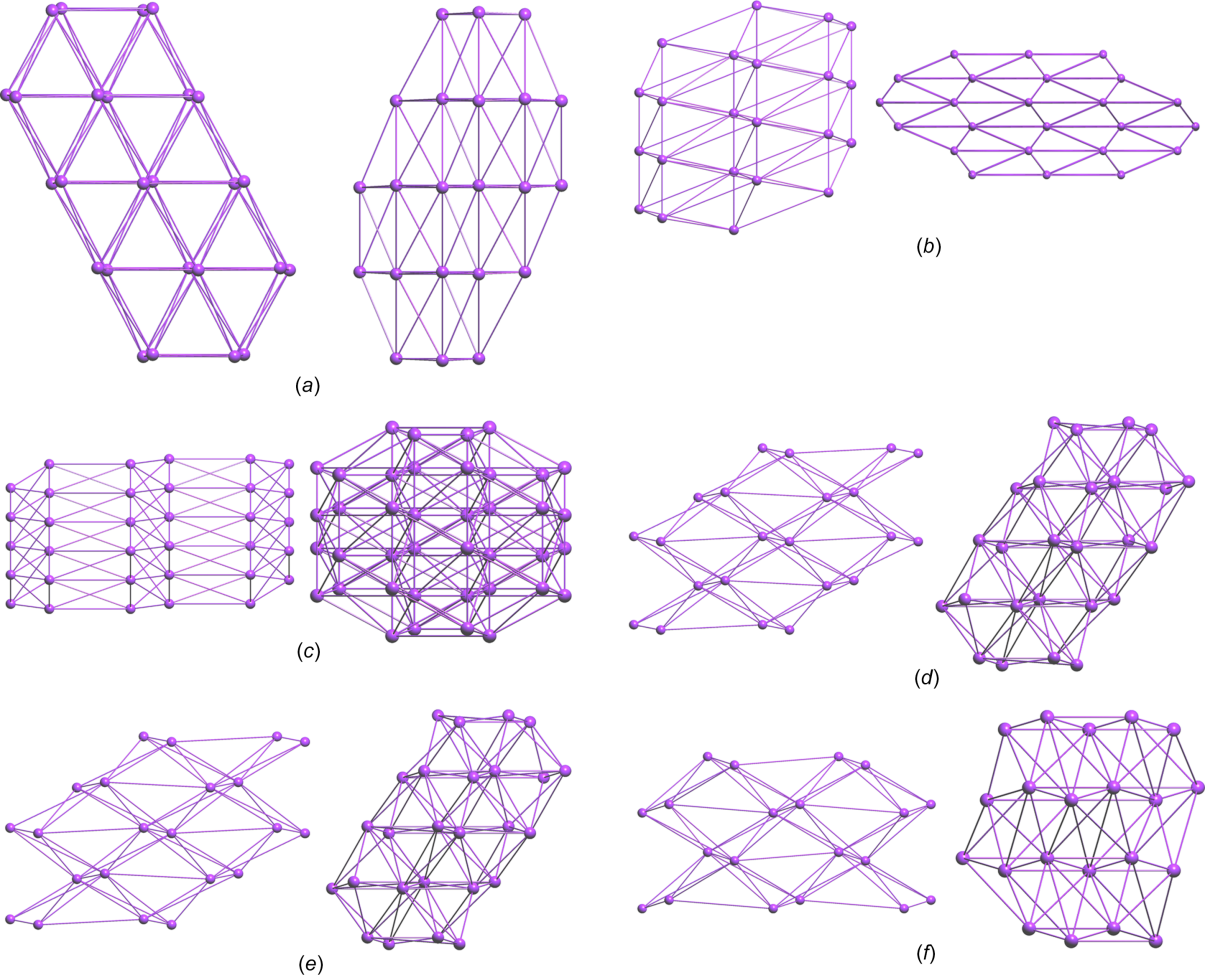
Topology networks determined with *TopCryst*. (*a*) **S1a** viewed down the *a* and *c* axes; (*b*) **S2a** viewed down the *b* and *c* axes; (*c*) **S3a** viewed down the *a* and *c* axes; (*d*) **S1b** viewed down the *a* and *c* axes; (*e*) **S2b** viewed down the *a* and *c* axes; (*f*) **S3b** viewed down the *a* and *c* axes.

Fig. 8 ▸ com­pares the relative percentage contributions of close contacts to the Hirshfeld surfaces for all the structures. As can be seen, the contributions of the O⋯H/H⋯O and S⋯H/H⋯S contacts together form a significant part of the inter­molecular inter­actions. Also indicated is the presence of O⋯C/C⋯O, C⋯C and C⋯H/H⋯C inter­actions in all the structures. Table 8 ▸ lists the lattice energies calculated with *CrystalExplorer*, taking into account all mol­ecules within a 20 Å radius. The energy framework diagrams (Fig. 6 ▸) show that all the structures have a three-dimensional framework. *TopCryst* and *Topospro* (Shevchenko & Blatov, 2021 ▸; Shevchenko *et al.*, 2022 ▸) can be used to determine the underlying topology network of all the intra­molecular inter­actions and provide a convenient way to describe the inter­action network. The network of the electrostatic and van der Waals inter­actions determined with *TopCryst* are shown in Fig. 9 ▸. All the networks determined for the structures are three-dimensional and correspond with the energy frameworks determined by *CrystalExplorer*.

## Conclusion

A number of novel coumarin derivatives were successfully synthesized and characterized. The inter­molecular inter­actions were investigated extensively using the energy framework feature of *CrystalExplorer*. This proved particularly useful for locating π-type inter­actions to better understand the total energy network, more so than what can be obtained by just looking at conventional inter­molecular hy­dro­gen-bond inter­actions. Furthermore, the topology of the different energy frameworks can be conveniently com­pared using *TopCryst*. The coumarin derivatives synthesized here all show extensive π-inter­actions in their structures. This, together with preliminary absorption spectrometry, indicate that the com­pounds are well suited for further investigations as chemosensors.

## Supplementary Material

Crystal structure: contains datablock(s) S1a, S2a, S3a, S1b, S2b, S3b, global. DOI: 10.1107/S2053229625000956/ef3062sup1.cif

Structure factors: contains datablock(s) S1a. DOI: 10.1107/S2053229625000956/ef3062S1asup2.hkl

Structure factors: contains datablock(s) S2a. DOI: 10.1107/S2053229625000956/ef3062S2asup3.hkl

Structure factors: contains datablock(s) S3a. DOI: 10.1107/S2053229625000956/ef3062S3asup4.hkl

Structure factors: contains datablock(s) S1b. DOI: 10.1107/S2053229625000956/ef3062S1bsup5.hkl

Structure factors: contains datablock(s) S2b. DOI: 10.1107/S2053229625000956/ef3062S2bsup6.hkl

Structure factors: contains datablock(s) S3b. DOI: 10.1107/S2053229625000956/ef3062S3bsup7.hkl

Supporting information file. DOI: 10.1107/S2053229625000956/ef3062S1asup8.cml

Supporting information file. DOI: 10.1107/S2053229625000956/ef3062S2asup9.cml

Supporting information file. DOI: 10.1107/S2053229625000956/ef3062S3asup10.cml

Supporting information file. DOI: 10.1107/S2053229625000956/ef3062S1bsup11.cml

Supporting information file. DOI: 10.1107/S2053229625000956/ef3062S2bsup12.cml

Supporting information file. DOI: 10.1107/S2053229625000956/ef3062S3bsup13.cml

CCDC references: 2421185, 2421184, 2421183, 2354585, 2354584, 2354583

## Figures and Tables

**Figure 4:**
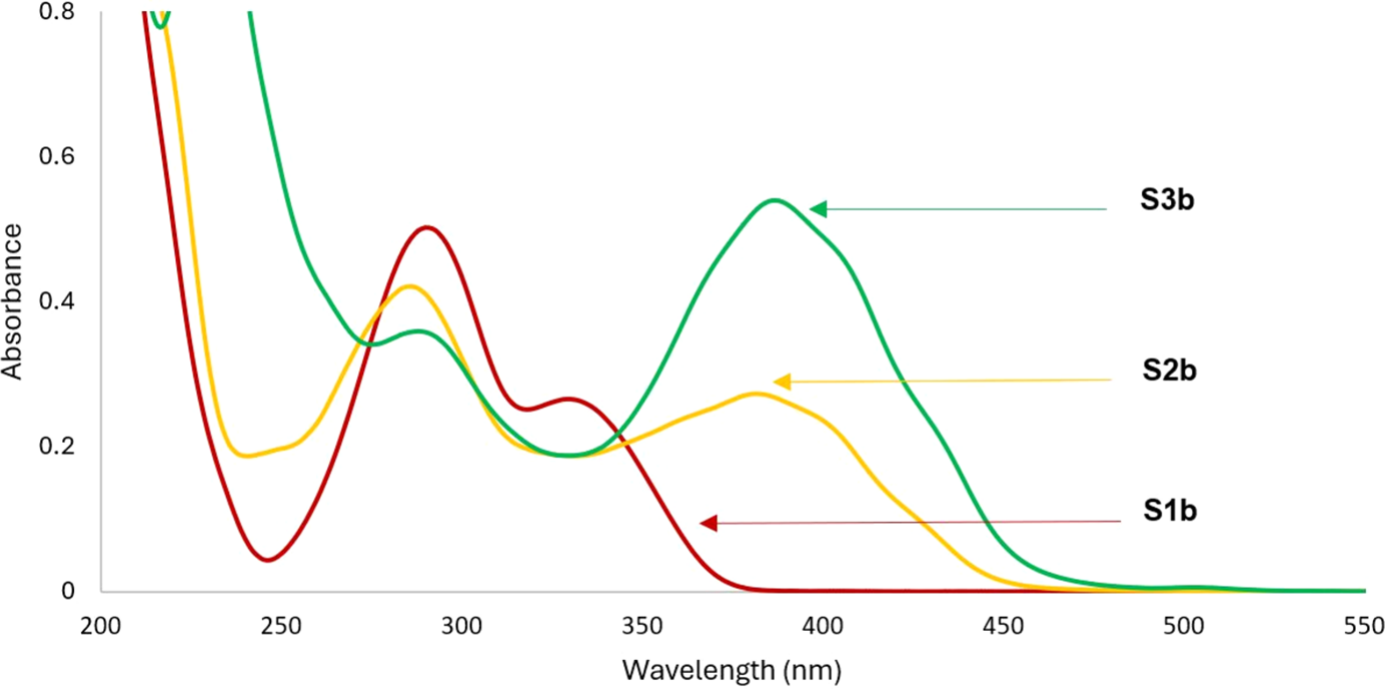
UV–Vis spectra of com­pounds **S1b**, **S2b** and **S3b** in aceto­nitrile medium.

**Table:** Experiments were carried out with Mo *K*α radiation. H-atom parameters were constrained. **S3b** was refined as an inversion twin [0.28 (16)].

	**S1a**	**S2a**	S**3a**
Crystal data			
Chemical formula	C_12_H_10_O_4_	C_12_H_10_O_3_S	C_12_H_10_O_2_S_2_
*M*_r_	218.20	234.26	250.32
Crystal system, space group	Monoclinic, *P*2_1_/*c*	Monoclinic, *P*2_1_/*c*	Monoclinic, *C*2/*c*
Temperature (K)	296	200	200
*a*, *b*, *c* (Å)	7.9043 (7), 15.7768 (13), 8.7381 (7)	11.9040 (9), 7.1792 (5), 13.6794 (10)	12.5314 (5), 6.7175 (3), 27.7746 (10)
α, β, γ (°)	90, 108.115 (4), 90	90, 111.708 (3), 90	90, 92.7398 (14), 90
*V* (Å^3^)	1035.67 (15)	1086.15 (14)	2335.38 (16)
*Z*	4	4	8
μ (mm^−1^)	0.11	0.29	0.44
Crystal size (mm)	1.00 × 0.75 × 0.44	0.87 × 0.66 × 0.58	0.30 × 0.16 × 0.11

Data collection			
Diffractometer	Bruker APEXII CCD	Bruker APEXII CCD	Bruker D8 QUEST
Absorption correction	Numerical (*SADABS*; Bruker, 2012 ▸)	Numerical (*SADABS*; Bruker, 2012 ▸)	Numerical (*SADABS*; Krause *et al.*, 2015 ▸)
*T*_min_, *T*_max_	0.950, 1.000	0.941, 1.000	0.891, 1.000
No. of measured, independent and observed [*I* > 2σ(*I*)] reflections	19039, 2575, 2036	18714, 2710, 2368	27927, 2894, 2326
*R*_int_	0.018	0.017	0.048
(sin θ/λ)_max_ (Å^−1^)	0.667	0.668	0.667

Refinement			
*R*[*F*^2^ > 2σ(*F*^2^)], *wR*(*F*^2^), *S*	0.050, 0.141, 1.05	0.035, 0.105, 1.04	0.040, 0.100, 1.10
No. of reflections	2575	2710	2894
No. of parameters	146	146	146
Δρ_max_, Δρ_min_ (e Å^−3^)	0.35, −0.17	0.36, −0.26	0.30, −0.29

**Table:** 

	**S1b**	**S2b**	**S3b**
Crystal data			
Chemical formula	C_13_H_12_O_5_	C_13_H_12_O_4_S	C_13_H_12_O_3_S_2_
*M*_r_	248.23	264.29	280.35
Crystal system, space group	Monoclinic, *P*2_1_/*n*	Monoclinic, *P*2_1_/*n*	Orthorhombic, *P*2_1_2_1_2_1_
Temperature (K)	200	199	200
*a*, *b*, *c* (Å)	6.8708 (3), 10.6766 (5), 15.7872 (8)	6.8534 (4), 11.2183 (7), 15.8581 (10)	6.9815 (3), 11.7185 (4), 15.9642 (5)
α, β, γ (°)	90, 100.253 (2), 90	90, 98.620 (2), 90	90, 90, 90
*V* (Å^3^)	1139.60 (9)	1205.45 (13)	1306.07 (8)
*Z*	4	4	4
μ (mm^−1^)	0.11	0.27	0.40
Crystal size (mm)	1.17 × 0.83 × 0.51	0.67 × 0.62 × 0.50	0.48 × 0.15 × 0.15

Data collection			
Diffractometer	Bruker APEXII CCD	Bruker APEXII CCD	Bruker D8 QUEST
Absorption correction	Numerical (*SADABS*; Bruker, 2012 ▸)	Numerical (*SADABS*; Bruker, 2012 ▸)	Numerical (*SADABS*; Krause *et al.*, 2015 ▸)
*T*_min_, *T*_max_	0.949, 1.000	0.938, 1.000	0.508, 1.000
No. of measured, independent and observed [*I* > 2σ(*I*)] reflections	20764, 2822, 2493	20324, 2981, 2607	39191, 3236, 2716
*R*_int_	0.023	0.021	0.091
(sin θ/λ)_max_ (Å^−1^)	0.667	0.667	0.666

Refinement			
*R*[*F*^2^ > 2σ(*F*^2^)], *wR*(*F*^2^), *S*	0.035, 0.099, 1.07	0.035, 0.104, 1.04	0.055, 0.141, 1.24
No. of reflections	2822	2981	3236
No. of parameters	166	165	166
Δρ_max_, Δρ_min_ (e Å^−3^)	0.32, −0.21	0.40, −0.32	0.66, −0.43

Computer programs: *APEX2* (Bruker, 2012 ▸), *SAINT* (Bruker, 2012 ▸), *SHELXT2018* (Sheldrick, 2015*a* ▸), *SHELXL2019* (Sheldrick, 2015*b* ▸), *ShelXle* (Hübschle *et al.*, 2011 ▸), *ORTEP-3 for Windows* (Farrugia, 2012 ▸), *PLATON* (Spek, 2020 ▸) and *Mercury* (Macrae *et al.*, 2020 ▸).

**Table 2:** Hydrogen-bond geometry (Å,°) for **S1a**[Chem scheme1]

*D*—H⋯*A*	*D*—H	H⋯*A*	*D*⋯*A*	*D*—H⋯*A*
C3—H3⋯O3	0.93	2.38	2.7074 (18)	101
C6—H6⋯O2^i^	0.93	2.72	3.352 (2)	126
C7—H7⋯O4^i^	0.93	2.72	3.647 (2)	175
C8—H8⋯O2^ii^	0.93	2.68	3.5281 (19)	153
C11—H11*B*⋯O4^iii^	0.97	2.55	3.335 (2)	138

Symmetry codes: (i) 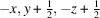; (ii) 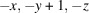; (iii) 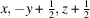.

**Table 3:** Hydrogen-bond geometry (Å,°) for **S2a**[Chem scheme1]

*D*—H⋯*A*	*D*—H	H⋯*A*	*D*⋯*A*	*D*—H⋯*A*
C3—H3⋯S1^i^	0.95	2.72	3.6489 (12)	166
C5—H5⋯O1^i^	0.95	2.89	3.8188 (16)	167
C8—H8⋯O3^ii^	0.95	2.54	3.4876 (19)	175
C12—H12*A*⋯S1^iv^	0.98	3.07	4.0287 (16)	168
C12—H12*B*⋯O3^iii^	0.98	2.69	3.567 (2)	150
C12—H12*B*⋯S1^ii^	0.98	3.26	4.137 (6)	151
C12—H12*C*⋯S1^i^	0.98	3.22	4.0821 (18)	148

Symmetry codes: (i) 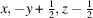; (ii) 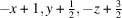; (iii) 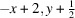, 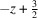; (iv) 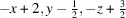.

**Table 4:** Hydrogen-bond geometry (Å,°) for **S3a**[Chem scheme1]

*D*—H⋯*A*	*D*—H	H⋯*A*	*D*⋯*A*	*D*—H⋯*A*
C3—H3⋯S1^i^	0.95	3.03	3.8486 (18)	146
C8—H8⋯O1^ii^	0.95	2.91	3.600 (2)	131
C12—H12*A*⋯S1^iii^	0.98	3.15	3.981 (2)	144
C12—H12*C*⋯S2^iv^	0.98	3.22	4.119 (3)	154
C12—H12*B*⋯S2^v^	0.98	3.33	4.245 (3)	156

Symmetry codes: (i) 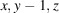; (ii) 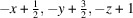; (iii) 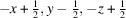; (iv) 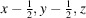; (v) 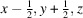.

**Table 5:** Hydrogen-bond geometry (Å,°) for **S1b**[Chem scheme1]

*D*—H⋯*A*	*D*—H	H⋯*A*	*D*⋯*A*	*D*—H⋯*A*
C5—H5⋯O4^i^	0.95	2.58	3.4065 (14)	146

Symmetry code: (i) 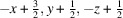.

**Table 6:** Hydrogen-bond geometry (Å,°) for **S2b**[Chem scheme1]

*D*—H⋯*A*	*D*—H	H⋯*A*	*D*⋯*A*	*D*—H⋯*A*
C3—H3⋯S1^i^	0.95	2.91	3.7460 (12)	147
C5—H5⋯O3^i^	0.95	2.59	3.4774 (17)	156
C11—H11*B*⋯O4^ii^	0.99	2.53	3.2803 (17)	132

Symmetry codes: (i) 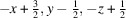; (ii) 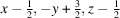.

**Table 7:** Hydrogen-bond geometry (Å,°) for **S3b**[Chem scheme1]

*D*—H⋯*A*	*D*—H	H⋯*A*	*D*⋯*A*	*D*—H⋯*A*
C5—H5⋯S2^i^	0.95	2.86	3.764 (4)	160
C11—H11*A*⋯O3^ii^	0.99	2.64	3.460 (6)	140
C11—H11*B*⋯S2	0.99	2.67	3.005 (5)	100
C12—H12*C*⋯S2^iii^	0.98	2.83	3.805 (7)	178
C13—H13*C*⋯S1^iv^	0.98	2.94	3.656 (5)	131

Symmetry codes: (i) 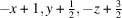; (ii) 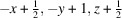; (iii) 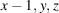; (iv) 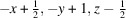.

**Table 8:** Calculated lattice energies (kJ mol^−1^)

**S1a**	−262	**S1b**	−326
**S2a**	−256	**S2b**	−318
**S3a**	−235	**S3b**	−295

## References

[bb1] Abdallah, M., Hijazi, A., Dumur, F. & Lalevée, J. (2020). *Molecules*, **25**, 2063. 10.3390/molecules25092063 PMC7248746 32354136

[bb2] Bakhtiari, G., Moradi, S. & Soltanali, S. A. (2014). *Arab. J. Chem.* **7**, 972–975.

[bb3] Bernstein, J., Davis, R. E., Shimoni, L. & Chang, N.-L. (1995). *Angew. Chem. Int. Ed. Engl.* **34**, 1555–1573.

[bb4] Bojtár, M., Kormos, A., Kis-Petik, K., Kellermayer, M. & Kele, P. (2019). *Org. Lett.* **21**, 9410–9414. 10.1021/acs.orglett.9b03624 31714093

[bb5] Bruker (2012). *APEX2*, *SAINT* and *SADABS*. Bruker AXS Inc., Madison, Wisconsin, USA.

[bb6] Farrugia, L. J. (2012). *J. Appl. Cryst.* **45**, 849–854.

[bb7] García-Báez, E. V., Martínez-Martínez, F. J., Höpfl, H. & Padilla-Martínez, I. I. (2003). *Cryst. Growth Des.* **3**, 35–45.

[bb8] Groom, C. R., Bruno, I. J., Lightfoot, M. P. & Ward, S. C. (2016). *Acta Cryst.* B**72**, 171–179. 10.1107/S2052520616003954 PMC4822653 27048719

[bb9] Hansen, M. J., Velema, W. A., Lerch, M. M., Szymanski, W. & Feringa, B. L. (2015). *Chem. Soc. Rev.* **44**, 3358–3377. 10.1039/c5cs00118h 25917924

[bb10] Hübschle, C. B., Sheldrick, G. M. & Dittrich, B. (2011). *J. Appl. Cryst.* **44**, 1281–1284. 10.1107/S0021889811043202 PMC3246833 22477785

[bb11] Jesberger, M., Davis, T. P. & Barner, L. (2003). *Synthesis*, pp. 1929–1958.

[bb12] Kayukova, L. A., Praliyev, K. D., Gut’yar, V. G. & Baitursynova, G. P. (2015). *Russ. J. Org. Chem.* **51**, 148–160.

[bb13] Khatoon, H. & Abdulmalek, E. A. (2021). *Molecules*, **26**, 6937.

[bb14] Krause, L., Herbst-Irmer, R., Sheldrick, G. M. & Stalke, D. (2015). *J. Appl. Cryst.* **48**, 3–10. 10.1107/S1600576714022985 PMC4453166 26089746

[bb15] Liu, X., Cole, J. M., Waddell, P. G., Lin, T. C., Radia, J. & Zeidler, A. (2012). *J. Phys. Chem. A*, **116**, 727–737. 10.1021/jp209925y 22117623

[bb16] Mackenzie, C. F., Spackman, P. R., Jayatilaka, D. & Spackman, M. A. (2017). *IUCrJ*, **4**, 575–587. 10.1107/S205225251700848X PMC5600021 28932404

[bb17] Macrae, C. F., Sovago, I., Cottrell, S. J., Galek, P. T. A., McCabe, P., Pidcock, E., Platings, M., Shields, G. P., Stevens, J. S., Towler, M. & Wood, P. A. (2020). *J. Appl. Cryst.* **53**, 226–235. 10.1107/S1600576719014092 PMC6998782 32047413

[bb18] Mahendra, M., Doreswamy, B. H., Sridhar, M. A., Prasad, J. S., Narodia, V. P., Naliapara, Y. T. & Shah, A. (2003). *Anal. Sci. X*, **19**, X33–X34.

[bb19] Matos, M. J., Santana, L., Uriarte, E., Abreu, O. A., Molina, E. & Yordi, E. G. (2015). *Phytochemicals – Isolation, Characterisation and Role in Human Health*, edited by A. V. Rao & L. G. Rao, ch. 5. London: IntechOpen. https://doi.org/10.5772/59982.

[bb20] Olson, J. P., Banghart, M. R., Sabatini, B. L. & Ellis-Davies, G. C. R. (2013). *J. Am. Chem. Soc.* **135**, 15948–15954. 10.1021/ja408225k PMC4097017 24117060

[bb21] Patil, S. A., Unki, S. N., Kulkarni, A. D., Naik, V. H. & Badami, P. S. (2011). *J. Mol. Struct.* **985**, 330–338.

[bb22] Shang, Y., He, X., Zhou, Y., Yu, Z., Han, G., Jin, W. & Chen, J. (2015). *Tetrahedron*, **71**, 864–868.

[bb23] Sheldrick, G. M. (2015*a*). *Acta Cryst.* A**71**, 3–8.

[bb24] Sheldrick, G. M. (2015*b*). *Acta Cryst.* C**71**, 3–8.

[bb25] Shevchenko, A. P. & Blatov, V. A. (2021). *Struct. Chem.* **32**, 507–519.

[bb26] Shevchenko, A. P., Shabalin, A. A., Karpukhin, I. Y. & Blatov, V. A. (2022). *Sci. Technol. Adv. Mater. Meth.* **2**, 252–265.

[bb27] Spackman, P. R., Turner, M. J., McKinnon, J. J., Wolff, S. K., Grimwood, D. J., Jayatilaka, D. & Spackman, M. A. (2021). *J. Appl. Cryst.* **54**, 1006–1011. 10.1107/S1600576721002910 PMC8202033 34188619

[bb28] Spek, A. L. (2020). *Acta Cryst.* E**76**, 1–11. 10.1107/S2056989019016244 PMC6944088 31921444

[bb29] Takahashi, H., Takechi, H., Kubo, K. & Matsumoto, T. (2006). *Acta Cryst.* E**62**, o2553–o2555.

[bb30] Tan, S. L., Jotani, M. M. & Tiekink, E. R. T. (2019). *Acta Cryst.* E**75**, 308–318. 10.1107/S2056989019001129 PMC6399703 30867939

[bb31] Zhang, G., Zheng, H., Guo, M., Du, L., Liu, G. & Wang, P. (2016). *Appl. Surf. Sci.* **367**, 167–173.

